# In Vitro Fermentation Characteristics of Purified Short-Chain Inulin and Inulin Neoseries Oligosaccharides Produced from Red Onions

**DOI:** 10.3390/foods14162804

**Published:** 2025-08-13

**Authors:** Jirat Wongsanittayarak, Apinun Kanpiengjai, Nalapat Leangnim, Supachawadee Soyprasert, Kridsada Unban, Saisamorn Lumyong, Chartchai Khanongnuch, Pairote Wongputtisin

**Affiliations:** 1 Program in Biotechnology, Multidisciplinary and Interdisciplinary School, Chiang Mai University, Chiang Mai 50200, Thailand; beerjirat.wong@gmail.com (J.W.); supachawadee_s@cmu.ac.th (S.S.); 2 Division of Biochemistry and Biochemical Innovation, Department of Chemistry, Faculty of Science, Chiang Mai University, Chiang Mai 50200, Thailand; nalapat.l@cmu.ac.th; 3 Office of Research Administration, Chiang Mai University, Chiang Mai 50200, Thailand; 4 Division of Food Science and Technology, Faculty of Agro-Industry, Chiang Mai University, Chiang Mai 50100, Thailand; kridsada.u@cmu.ac.th; 5 Department of Biology, Faculty of Science, Chiang Mai University, Chiang Mai 50200, Thailand; scboi009@gmail.com; 6 Academy of Science, The Royal Society of Thailand, Bangkok 10300, Thailand; 7 Center of Excellence in Agricultural Innovation for Graduate Entrepreneur, Maejo University, Chiang Mai 50290, Thailand; ck_biot@yahoo.com; 8 Program in Biotechnology, Faculty of Science, Maejo University, Chiang Mai 50290, Thailand; pairotewong@gmail.com

**Keywords:** red onions, fructooligosaccharides, neokestose, prebiotics, fecal fermentation

## Abstract

In our previous study, short-chain inulin and inulin neoseries oligosaccharides (SCIINOs) were produced and purified from red onion juice. This study aimed to investigate the effect of SCIINOs on changes in the bacterial composition of fecal microbiota obtained from normal weight, overweight, and obese subjects using in vitro batch fermentation. Fermentation characteristics, including changes in fecal microbiota determined by the V3–V4 region of 16S rRNA amplicon sequencing, residual SCIINO content, and the resulting organic acid profiles, were determined. The results indicate that SCIINOs were fermentable, which occurred along with a decrease in the SCIINO content and an increase in lactic, acetic, propionic, and butyric acids. The microbial composition of fecal inoculum influenced the degree of SCIINO fermentation, which was then associated with the fermentation outcomes. Alpha-diversity results revealed that fermentation with and without SCIINOs decreased species richness, evenness, and diversity. Beta-diversity results revealed that fermentation of SCIINOs using all fecal inocula negatively affected the abundance of *Escherichia-Shigella* and *Klebsiella* while positively affecting the abundance of *Lactococcus*. The enrichment of *Lactococcus* was confirmed by an independent study, indicating that two reference strains of *Lactococcus lactis* efficiently utilized neokestose and nystose as the major FOS constituent present in SCIINOs.

## 1. Introduction

Fructooligosaccharides (FOSs) are oligosaccharides of fructose that have a glucose molecule at their non-reducing end. They are typically found in certain plants such as Jerusalem artichokes and chicory roots [1]. To date, FOSs are considered the most acceptable and widely used prebiotic oligosaccharides [2], as they can be fairly easily dispersed across populations and enrich the beneficial bacteria in the human gut, particularly *Bifidobacterium* [3]. Short-chain FOSs (SCFOSs), such as kestose (GF_2_) and nystose (GF_3_), are the most competent FOSs, as they have been found to undergo more rapid fermentation by beneficial microorganisms than other long-chain FOSs [4,5]. In addition, there is another FOS structure called neo-FOS that naturally occurs at low levels in some plants, such as onions and garlic, and is present in the form of a mixture between inulin- and neo-FOSs [1]. Notably, neo-FOSs, a branched structure of FOSs, have been determined to be more tolerant of high temperatures and low pH conditions than inulin-FOSs [6]. Generally, when inulin-FOSs are produced under such conditions, they release simple sugars including fructose, glucose, and sucrose. Importantly, neo-FOSs could be applicable for thermal processing in the food industry.

Our previous study has revealed that the juice of red onions (*Allium cepa* var. *viviparum*) can serve as an alternative source of prebiotics. This juice mainly comprises inulin and inulin neoseries oligosaccharides that have the ability to encourage a bifidogenic effect, specifically in terms of the growth of *Bifidobacterium* (*B*.) *breve* and other lactobacilli probiotics [7]. This finding is in accordance with the outcomes of a previous review report that comprised a systematic review and a meta-analysis that included an evaluation of the effects of FOS supplementation on human fecal microbiota [3]. Moreover, the red onion juice was found to contain simple sugars (fructose, glucose, and sucrose) that may have reduced any potential prebiotic properties [7,8]. When consuming a simple sugar-containing prebiotic at a certain dosage, it is important to note that the amount of prebiotic consumed would be lower than if the prebiotic was consumed alone. A large amount of simple sugars can be absorbed by the upper gastrointestinal system before they reach the large intestine, which is indicative of one of the risk factors for developing non-communicable diseases (NCDs) [9]. When consuming a high sugar diet, simple sugars can reach the large intestines where they are non-selectively fermented and can alter the gut environment that is linked to the development of diseases associated with gut microbiota dysbiosis such as colitis [10]. Therefore, attempts have been made to produce short-chain inulin and inulin neoseries oligosaccharides (SCIINOs), along with the removal of simple sugars from the produced SCIINOs. Accordingly, the red onion juice was partially hydrolyzed by endo-inulinase to produce the SCIINOs, while *Candida orthopsilosis* FLA44.2 was used to remove simple sugars from the SCIINOs via the fermentation approach. Neokestose has been identified as a major FOS constituent of SCIINOs, whereas the others were identified as short-chain inulin-FOSs [11]. From our point of view, SCIINOs could potentially exert certain positive effects on the selective stimulating growth of beneficial bacteria apart from *Bifidobacterium*, which is known to be stimulated by various inulin- and neo-FOSs [12]. This attribute suggests that SCIINOs could be used for the prevention and/or treatment of diseases associated with gut microbiota dysbiosis, particularly obesity. This intestinal dysbiosis can contribute to various metabolic disorders such as heart disease, diabetes, and liver disease [13]. This study aimed to investigate the effect of SCIINOs on changes in the bacterial composition of fecal microbiota obtained from normal weight, overweight, and obese subjects via in vitro batch fermentation and to determine the relevant fermentation characteristics in terms of the fermentability of SCIINOs and the profiles of lactic, acetic, propionic, and butyric acids produced during SCIINO fermentation. This would increase the research interest into utilizing SCIINOs produced from red onions as a functional gradient. In addition, the possible mechanisms that serve as the most abundant bacteria to utilize SCIINOs were also discussed.

## 2. Materials and Methods

### 2.1. Preparation of Short-Chain Inulin and Inulin Neoseries Oligosaccharides

Red onions (*Allium cepa* L. var. *viviparum*) were purchased from the Muang Mai Market, Muang District, Chiang Mai, Thailand in May of 2022. Red onion juice, as the source of short-chain inulin and inulin neoseries oligosaccharides (SCIINOs), was prepared using the method described in our previous study [11]. Briefly, red onions were chopped into 3 × 3 cm pieces for the extraction of juice using an JT-2010 Healthy Slow Juicer (Jutian, Foshan, China). The juice was determined for total fructan content using a Fructan HK assay kit (Megazyme, Wicklow, Ireland). The quantified fructan content was used for calculating the specific amount of endo-inulinase (Creative-Enzyme^®^, Shirley, NY, USA) to be used for SCIINO production. An optimal initial concentration of endo-inulinase was determined to be 0.4 U/g of the initial fructan content present in red onion juice. *Candida orthopsilosis* FLA44.2 was used for the fermentative removal of residual simple sugars present in red onion juice, as well as those released via the catalytic activity of endo-inulinase. To apply the yeast for production and purification of SCIINOs, a total of 5% (*v*/*v*) of 24 h yeast culture cultivated at 30 °C on a 120 rpm rotary shaker was centrifuged at 6000 rpm, and at 20 °C for 10 min to collect the cell pellets. To produce SCIINOs, along with the selective removal of simple sugars, the red onion juice was sterilized via autoclaving at 121 °C for 15 min. After cooling, it was supplemented with 0.4 U of endo-inulinase/g total fructans and the freshly generated cell pellets of *C*. *orthopsilosis* FLA44.2. The fermentation process was carried out on a 120 rpm rotary shaking incubator at 30 °C. After 72 h of cultivation, SCIINOs were terminated by being heated to 80 °C for 30 min and left to cool. The resulting culture was centrifuged to collect a SCIINO solution. Subsequently, HPLC was used to confirm that the SCIINOs were then free from residual absorbable sugars including fructose, glucose, and sucrose. The purified solution was demineralized by Amberlite^®^ MB20 mixed ion exchange resin (Sigma Aldrich, Darmstadt, Germany) at 20 °C. After 4 h of applying the demineralization step, the resin was removed by filtering the solution through a sheet cloth. The achieved solution was decolorized and deodorized by an activated charcoal (Sigma Aldrich, Darmstadt, Germany) column to remove the color and odor of red onions [14]. Total FOSs were determined using a Fructan HK assay kit. Accordingly, this purified SCIINO consisted of neokestose (neo-GF_2_), kestose (GF_2_), nystose (GF_3_), fructofuranosylnystose (GF_4_), and other longer chain FOSs at levels of 2.77 ± 0.05, 0.33 ± 0.00, 1.72 ± 0.21, 1.81 ± 0.04, and 3.34 ± 0.16 g/L, respectively. The structures of these FOSs are presented in Figure S1. No fructose, glucose, or sucrose was detected. The SCIINOs were then passed through a 0.22 µm sterile membrane filter prior to being used for in vitro fecal fermentation experiments.

### 2.2. Volunteer Recruitment and Fecal Sample Collection

With regard to their alternative weight status classification, or the World Health Organization’s Regional Office (WPRO) standard, subjects who were determined to possess biomass index (BMI) values of 18.5–22.9, 23–24.9, and ≥25.0 could be classified as normal weight (NO), overweight (OV), and obese (OB) donors, respectively [15]. To investigate the fermentability effect of SCIINOs when fermented with different fecal samples as a natural mixed culture, study subjects comprised three healthy Thai adults who were either employees or graduate students at the Faculty of Science, Chiang Mai University, Chiang Mai, Thailand. The normal weight donor was a 30-year-old male with a BMI of 22.8, the overweight donor was a 32-year-old male with a BMI of 24.8, and the obese donor was a 30-year-old male with a BMI of 27.8. It was required that the donors must have had no record of receiving antibiotics for at least one month, and that there was no evidence of visible or detectable diseases prior to the collection of their fecal samples. The study protocol was approved by the Research Ethics Committee (CMUREC No. 66/087, date: 26 May 2023) of Chiang Mai University, Chiang Mai, Thailand, and the methods were performed in accordance with the relevant guidelines and regulations. The recruited donors were asked to sign an informed consent form before being enrolled in this study. Fecal samples were collected in sterile plastic containers immediately after defecation in the early morning, placed in an anerobic jar, kept on ice, and transported to the laboratory within 30 min. No fasting was required of the donors.

### 2.3. In Vitro Fecal Batch Fermentation

Upon arrival, each fecal sample was prepared for use as an inoculum by dilution with phosphate buffer saline (PBS) to yield a final concentration of 10% (*w*/*v*), which was then used as the fecal inoculum. The culture medium used in this study was a standard basal medium used for in vitro fecal batch fermentation. It had been developed based on the basic requirements for large intestinal microbial growth [16]. In this study, each ingredient was purchased from HiMedia (Nashik, India) and Sigma Aldrich (Darmstadt, Germany). Accordingly, the basal medium consisted of 2 g/L peptone, 2 g/L yeast extract, 0.1 g/L NaCl, K_2_HPO_4_ 0.04 g/L, 0.04 g/L KH_2_PO_4_, 0.01 g/L MgSO_4_·7H_2_O, 0.01 g/L CaCl_2_·7H_2_O, 2 g/L tween80, 0.02 g/L hemin, 10 mL/L vitamin K1, 0.5 g/L cysteine HCl, and 0.5 g/L bile salts. Additionally, 10 g/L of total FOSs, which was determined with a total fructan assay kit for the specified treatment with and without the supplementation, was used as the control. The medium was adjusted to a pH of 7.0 and was then filtered through a sterile 0.22 µm filter before being used. Fecal fermentation was performed by transferring a total of 1% (*v*/*v*) of the inoculum to a laboratory bottle containing 100 mL of basal medium. Each bottle was administered with a sterile gas mixture of N_2_:CO_2_:H_2_ at a ratio of 80:10:10 (*v*/*v*/*v*) for 10 min. The bottles were then incubated at 37 °C on a 50 rpm rotary shaker. Samples were collected at 0, 12, and 24 h of the cultivation process. The gas mixture was compressed into the fermentation system after sample collection to bring about an anerobic state. Fecal pellets were separated from the culture broth by centrifugation at 12,000 rpm at 4 °C for 15 min. The obtained pellets were then used for further DNA extraction, while the supernatant was used in the determination of glucose, fructose, sucrose, and detectable SCIINO contents, which included neo-GF_2_, GF_2_, GF_3_, and GF_4_, by high performance liquid chromatography (HPLC). The relevant pH values and quantities of lactic acid, acetic acid, propionic acid, and butyric acid were also determined by HPLC. Overall, there were 15 samples, as presented in Table 1. The experiment was then independently performed in triplicate.

### 2.4. Extraction of Genomic DNA and 16S rRNA Gene Amplicon Sequencing

Total genomic DNA of each sample was extracted using a TIANamp Stool DNA kit (Tiangen Biotech, Beijing, China) according to the manufacturer’s instructions. Degradation of DNA was observed by agarose gel electrophoresis. DNA quality and quantity were evaluated using the spectrophotometric method. A ratio of A_260_/A_280_ within a range of 1.8 to 2.0, along with a minimum amount of 2.0 µg, was considered optimum. For library construction, PCR amplification of the V3–V4 region of the 16S rRNA gene was performed by employing specific primer connecting barcodes. PCR products of proper size (not exceeding 500 bp) were selected through 2% (*w*/*v*) agarose gel. To prepare the library for sequencing, an equivalent amount of the PCR products from each sample was combined. They were repaired at their ends and an adenine (A) base was added before they were ligated with Illumina sequencing adapters. The library was quantified by real-time PCR and determined for size distribution by a bioanalyzer. After that, quality control passed libraries were pooled and sequenced on Illumina platforms (Illumina, San Diego, CA, USA). This step was facilitated by Novogene Bioinformatics Technology (Beijing, China).

### 2.5. Bioinformatic Analysis

FLASH version 1.2.11 was used to merge paired-end reads to achieve raw tags [17], which were subsequently filtered using fastp software version 0.23.1 [18]. The tags obtained were clean and were of high quality. Upon sequence alignment administered by the Silva database, the chimera sequences were detected and removed by Vsearch package version 2.16.0 [19] to achieve the effective tags. Amplicon sequence variants (ASVs) were obtained after the effective tags had been denoised with the DADA2 module in the QIIME2 software version QIIME2-202202 [18]. For rapid multiple sequence comparisons, QIIME2 software based on the Silva 138.1 database was used for species annotation. The absolute abundance of ASVs was normalized and further used for alpha-diversity and beta-diversity analyses.

Alpha-diversity, performed based on the derived ASVs using QIIME2 software, was used to analyze the richness, evenness, and diversity of the initial fecal inoculum (NOC0, OVC0, and OBC0) and those characteristics that were present in the samples collected from fermentation with and without SCIINOs. Herein, observed features, Chao1, and dominance indices were selected to identify richness, while evenness was identified by applying the Pielou index. Shannon and Simpson indices were selected to identify community diversity, while the Coverage index was used to calculate sequencing depth.

To evaluate the complexity of the community composition and compare the differences between groups, the beta-diversity of all samples was calculated based on the weighted UniFrac distance in QIIME2. A cluster tree was constructed using the unweighted pair group method with the arithmetic mean (UPGMA), which was based on the weighted UniFrac distance matrix. A UPGMA diagram was drawn through the upgma.tre function within QIIME. Principal coordinate analysis (PCoA) was performed to achieve principal coordinates and to visualize the results from complex and multidimensional data. PCoA analysis was administered by ade4 package and ggplot2 package in R software version 4.0.3. Notably, LEfSe is a software package designed to discover high-dimensional biomarkers and reveal metagenomic characteristics. It emphasizes statistical significance, biological consistency, and effect correlation [20]. Herein, the non-parametric factorial Kruskal–Wallis (KW) sum-rank test was first applied to detect features with significant differential abundance pertaining to the class of interest. Then, biological consistency was investigated using a set of pairwise tests among the sub-class using the Wilcoxon rank-sum test. Finally, the result was expressed as a histogram of LDA scores to estimate the effect size of each differentially abundant feature. A threshold of greater than 4 was considered a significant effect size. Subsequently, LEfSe was analyzed by an exclusive package, namely the lefse version 1.1.01. LEfSe was used to discover biomarkers among NOC0-OBC0-OVC0, NOT12-OBT12-OVT12, NOT24-OBT24-OVT24, NOC0-NOC12-NOT12, OVC0-OVC12-OVT12, and OBC0-OBC12-OBT12. The *t*-test was performed to determine microorganisms that exhibited significant variations between groups (*p* < 0.05) at various taxonomic ranks. The results were analyzed using R software version 4.0.3. The Benjamini–Hochberg method was used for multiple test correction of the *p*-value. Significance was considered when *p* < 0.05, yet the significant threshold was *q* < 0.05 [21]. The *t*-test was used to determine the bacterial genus that exhibited significant changes between samples NOC12-NOT12, OVC12-OVT12, OBC12-OBT12, NOC0-NOT12, NOC0-NOC12, OVC0-OVT12, OVC0-OVC12, OBC0-OBT12, and OBC0-OBC12.

### 2.6. High Performance Liquid Chromatography

Lactic acid, acetic acid, propionic acid, and butyric acid were determined using the Rezex ROA-organic acid H+ (8%) column as the stationary phase and 2.5 mM sulfuric acid as the mobile phase. Flow rate and separation temperature were set up at 0.5 mL/min and 40 °C, respectively. Separated organic acids were detected by UV-Vis detector at 210 nm absorbance. The content was then calculated and expressed as g/L. Standard organic acids were purchased from Sigma-Aldrich (St. Louis, MO, USA).

A Shodex Asahipak (NH_2_P-40 4E) column was used to determine the contents of fructose, glucose, and sucrose. The column was equilibrated with the mobile phase consisting of acetonitrile and deionized water at a ratio of 75:25 (*v*/*v*). To separate each sugar, the flow rate and temperature were set up at 0.8 mL/min and 30 °C, respectively. The separated sugars were then detected by refractive index detector (RID). For analysis of neo-GF_2_, GF_2_, GF_3_, and GF_4_ contents, the Shodex HILICpak (VN-50 4D) column was equilibrated with a mobile phase consisting of acetonitrile and deionized water at a ratio of 80:20 (*v*/*v*). Separation conditions and detection of the oligosaccharides were achieved using the same separation conditions described above. Standard fructooligosaccharides (FOSs), including GF_2_, GF_3_, and GF_4_, were purchased from Wako Chemical (Osaka, Japan). Neo-GF_2_ was produced by *Xanthophyllomyces dendrorhous* TISTR 5730 and was purified by applying the chromatographic method with some modifications [22].

### 2.7. Determination of the Ability of Lactococcus sp. In Fermentation of Neokestose, 1-Kestose, and Nystose

*Lactococcus* (*Lc*.) *lactis* FS38.4 isolated from pickled bamboo shoots [23], and *Lc*. *lactis* TISTR 1401 derived from the Thailand Institute of Scientific Technological Research (TISTR), were used as reference strains for determination of the ability to ferment short-chain FOSs. Briefly, initial concentrations of 10^6^ cells/mL were cultivated at 37 °C in deMan Rogosa and Sharpe (MRS) broth containing a mixture of pure neo-GF_2_ (10 g/L), GF_2_ (10 g/L), and GF_3_ (10 g/L). During the fermentation process, samples were taken at 0, 6, 12, 24, 36, and 48 h to measure optical density at 600 nm. A portion of the samples taken were centrifuged at 12,000 rpm for 5 min to separate cells from the clear supernatant. The clear supernatant was then used for the further determination of residual neo-GF_2_, GF_2_, and GF_3_, and was also used as an extracellular fraction. The cells were washed twice with 20 mM sodium phosphate buffer at a pH of 6.5. They were then resuspended in the same buffer and used as an intact cell fraction. A portion of the cell suspension was disrupted by sonication for 15 min. The cell extract was then used as an intracellular fraction. All fractions were assayed for β-fructofuranosidase activity according to our previously described methods [14].

### 2.8. Statistical Analysis

Full factorial complete randomized design (CRD) was used to compare differences between the mean values of SCIINOs and the SCFA contents. When there were significant differences (*p* < 0.05), multiple comparison tests were performed based on all pairwise comparisons using Tukey’s HSD test at a confidence level of 95%. Statistix software version 8.0 (Analytical software, Tallahassee, FL, USA) was used for design and analysis.

## 3. Results and Discussion

### 3.1. Fermentability of Short-Chain Inulin and Inulin Neoseries Oligosaccharides

**Figure 1:**
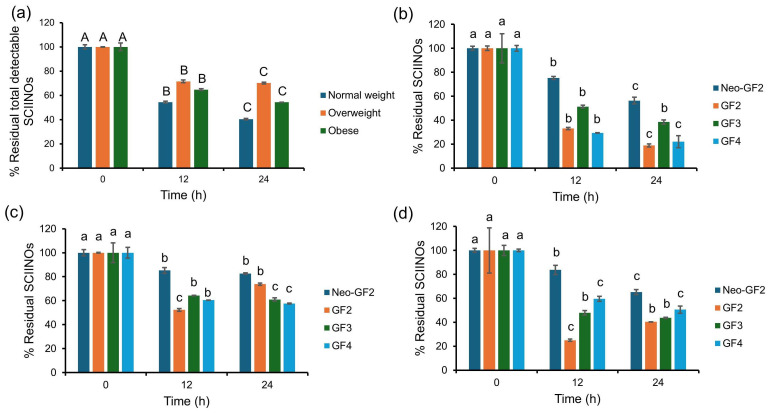
Percentage of residual total detectable SCIINOs during the in vitro fecal fermentation (**a**) and percentage of each residual detectable SCIINO content during fermentation using fecal inocula obtained from normal weight (**b**), overweight (**c**), and obese (**d**) subjects. Uppercase letters indicate significant differences (*p* < 0.05) of each subject. Lowercase letters indicate significant differences (*p* < 0.05) of each detectable SCIINO in different samples.

Fermentation is defined as a process that utilizes microorganisms to convert substrates into various products [24]. This is in accordance with the outcomes of this study, which revealed that the total detectable SCIINO content was significantly reduced to approximately 40–60% of the residual total SCIINO content within 12 h of fermentation. However, this gradually decreased until 24 h of fermentation (Figure 1a). Accordingly, both lactic acid and SCFAs, as the resulting products, increased (see Section 3.4). The decrease in SCIINO utilization may have been due to the accumulation of metabolites, specifically lactic acid and SCFAs, that could have inhibited the growth of some gut microorganisms. This phenomenon was also observed in the in vitro experiments of a previous study [25]. At 12 h of fermentation, fecal inoculum obtained from the normal weight subject exhibited an ability for significant utilization of the neo-GF_2_, GF_3_, and GF_4_ (Figure 1b) present in SCIINOs, and their residual contents were significantly decreased until the end of the fermentation process. On the other hand, the fermentation system using fecal inoculum of the overweight and obese subjects exhibited different profiles of SCIINO utilization (Figure 1c,d) from that of the normal weight subject. Herein, it was found that both fermentation systems revealed a decrease in neo-GF_2_, GF_3_, and GF_4_ contents, while the GF_2_ content significantly increased at the end of the fermentation process. Moreover, it was found that the residual content of GF_2_ significantly increased in accordance with a decrease in the residual contents of GF_3_ and GF_4_, as well as an elevation of residual fructose content (Figure S2). It could, therefore, be assumed that a decrease in the residual content of detectable SCIINOs reported in the fermentation processes using the fecal inoculum obtained from overweight and obese subjects appeared with both fermentation and degradation effects. Mechanisms that contribute to FOS utilization by gut microbes involve carbohydrate-active enzymes (CAZymes), transport systems, and complex microbial interactions. These mechanisms require steps to generate fructose from FOSs for microbial metabolisms [26]. As has been reported in previous studies, the bacterial composition of fecal samples obtained from the normal weight subject is more diverse and has higher richness than those obtained from overweight and obese subjects [27,28]. In addition, it commonly consists of beneficial bacteria that can normalize the dysbiosis of gut microbiota and thereby regulate risk factors associated with obesity and various metabolic syndromes [29]. Most beneficial bacteria are capable of effectively utilizing various prebiotics and producing beneficial metabolites that exhibit inhibitory activity against bacterial pathogens [30]. Based on the residual content of the detectable SCIINOs, it was clearly demonstrated that fecal inoculum obtained from the normal weight subject exhibited higher efficacy in the fermentation of SCIINOs than that which was derived from both overweight and obese subjects.

### 3.2. Changes in Bacterial Composition and Diversity

**Figure 2:**
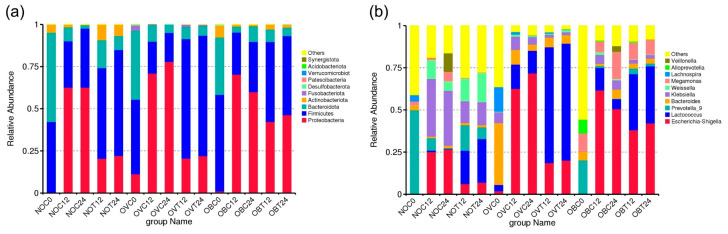
Relative abundance of the bacterial community at the top 10 phylum (**a**) and genus (**b**) levels during the in vitro fecal fermentation with SCIINOs (NOT12, NOT24, OVT12, OVT24, OBT12, and OBT24) and without SCIINOs (NOC0, NOC12, NOC24, OVC0, OVC12, OVC24, OBC0, OBC12, and OBC24).

The data processing statistics and quality control results for the V3–V4 amplicon sequencing are presented in Table S1. An average number of 174,397 ± 16,048 tags with an average length of 425.8 bp were obtained with the maximum and minimum numbers of 174,397 and 132,200 tags, respectively. After the noise reduction procedure, tag sequences that exhibited 100% similarity were clustered, and each de-duplicated sequence was considered a called amplicon sequence variant (ASV) or a feature sequence [31]. After the species annotation of each ASV was determined based on the Silva database, the relative abundance results at the levels of kingdom, phylum, class, order, family, genus, and species were obtained. Herein, only the relative abundance at the levels of phylum and genus is illustrated in Figure 2, with those of the other bacterial levels being shown in Figure S3. As is typically found in the bacterial community present in the human intestines [32], the top three phyla of all groups were Proteobacteria, Firmicutes, and Bacteroidota. This information is presented with a note that there was low relative abundance of Proteobacteria detected in each fecal inoculum, particularly for the NOC0, OVC0, and OBC0 samples. The fecal microbiota abundance of healthy adults and infants are known to be relatively stable at the phylum level when compared with those of the older subjects, while those at the genus and species levels have been found to be variable depending upon geography, environment, diet, and age [33,34]. For people who have BMI values that indicate being overweight and/or obese, they have been recognized to have high abundance ratios of Firmicute to Bacteroidetes when compared with normal weight people [35,36], which was in accordance with the findings of this study. Fecal microbiota of the normal weight (NOC0), overweight (OVC0), and obese (OBC0) subjects were dominated by relative abundance values of 42%, 44%, and 57% for Firmicutes and 53%, 41%, and 34% for Bacteroidetes (Figure 2a), thus indicating F/B ratios of 0.79, 1.07, and 1.68, respectively. At the genus taxonomic rank, more than 80% of all ASVs could be classified within 30 genera (Figure S4). Fermentation with and without SCIINOs revealed *Escherichia-Shigella*, *Lactococcus*, *Prevotella 9*, *Bacteroides*, and *Klebsiella* as the top five genera among all assessed samples (Figure 2b). Variations in their relative abundance was clearly associated with the presence of SCIINOs and was suggested to be associated with oxygen requirements, as has been stated in a previous study [37]. Although in this study, a simulated gastrointestinal gas mixture was applied in each fermentation system, the actual conditions were not strictly anerobic because of limitations in the sampling procedure. According to an overview of the major relative abundance values of *Prevotella 9* and *Bacteroides*, two obligated anerobic bacterial genera [38], were found to be markedly declined by an increase in fermentation time. In contrast, *Escherichia-Shigella* [39], *Lactococcus* [40], and *Klebsiella* [41], recognized as facultative anerobic bacterial genera, exhibited high relative abundance during the fermentation process. When compared with the control samples (fermentation without SCIINOs), the fermentation of SCIINOs partially increased the relative abundance of *Escherichia-Shigella* and *Klebsiella*, but was dominated by the high relative abundance of *Lactococcus*, a group of lactic acid bacteria. It was noted that all the assessed control samples were dominated by *Escherichia-Shigella* and *Klebsiella*. The SCIINO fermentation characteristics were in relative accordance with the findings obtained from a previous study, which reported that the fecal fermentation of pectin fractions could increase the abundance of lactic acid bacteria, while inhibiting *Escherichia-Shigella*, *Enterococcus*, and *Klebsiella* [42]. These outcomes were also in agreement with the in vitro fermentation of partially hydrolyzed guar gum by fecal microbiota, notably in the fermentation that stimulated the growth of *Bifidobacterium* and *Faecalibacterium* but inhibited the growth of *Escherichia-Shigella* and *Klebsiella* [43].

**Figure 3:**
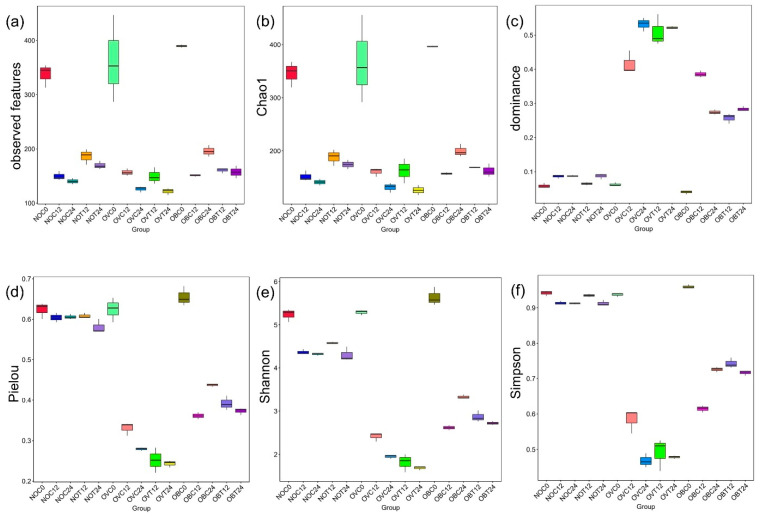
Box plots showing the richness (observed features (**a**) and Chao1 (**b**)) and the alpha-diversity (dominance (**c**), Pielou (**d**), Shannon (**e**), and Simpson (**f**) indices) values of bacterial communities in samples collected during the in vitro fecal fermentation process of SCIINOs, and in those experiments without SCIINOs using fecal inoculum obtained from normal weight, overweight, and obese subjects.

Alpha-diversity was applied to analyze microbial communities in terms of richness, evenness, and diversity [44]. The observed features index, or the observed species index (Figure 3a), implied that the number of species found in the assessed group excluded the singleton sequences. However, this index was used to assess the richness of each sample. The results of the index were determined to be in agreement with those of the Chao1 index (Figure 3b), which also implied that a number of species found in the assessed group included singleton sequences. Thus, the Chao1 index of each group was slightly higher than that of the observed features index when a few numbers of singleton sequences were found. These results are in accordance with a good coverage value of 1.0 obtained from all assessed groups (Figure S5). The dominance index (Figure 3c) was used to measure the dominance of one species over others within the same group. The higher value of the dominance index indicated the better homogeneity of the community species, but it indirectly reflected a lower degree of evenness. The Pielou index (Figure 3d) is a species evenness index that is used to measure the uniformity of microbial communities [45]. A higher value of the dominance index relates to a lower value of the Pielou index, which in turn reveals a low degree of evenness. The Shannon index determines the richness of the species and rare cover types, whilst the Simpson index emphasizes the evenness of the species and of the dominant cover types [46]. High Shannon and Simpson indices indicate a high degree of diversity present in the community. Our findings revealed that the Shannon index (Figure 3e) results for all samples were in accordance with the Simpson index (Figure 3f) results. 

Based on the six assessed alpha-diversity indices, the NOC0, OVC0, and OBC0 samples exhibited similar values of richness (Figure 3a,b), evenness (Figure 3c,d), and diversity (Figure 3e,f). Although some studies revealed that the fecal microbiota of the overweight and obese subjects could be associated with lower species richness, evenness, and diversity values than those of the normal weight subject [28,47], this statement is in conflict with the findings of this study. Previously, species richness and diversity values of fecal microbiota among normal weight and obese subjects had been discussed. Accordingly, a systematic review of gut microbiome in obese and non-obese subjects has been studied. The alpha-diversity results revealed that two of seven studies showed no significant differences in the Chao1 index results. Additionally, 11 of 22 studies indicated no significant differences in the Shannon index results, while 2 of 4 studies reported no significant differences in the Simpson index results [48]; yet, it seems likely that a definitive conclusion from these results cannot be drawn.

The fecal inoculum obtained from all subjects (NOC0, OVC0, and OBC0) exhibited a higher degree of species richness than for all the experimental treatments (NOT12, NOT24, OVT12, OVT24, OBT12, and OBT24) and the respective controls (NOC12, NOC24, OVC12, OVC24, OBC12, and OBC24). Yet, it was then determined that in vitro fermentation reduced the degree of species richness. In addition, it could be assumed that lower species richness was associated with a longer fermentation time. The species richness of the samples collected at 12 h of fermentation was likely higher than that which had been recorded after 24 h of fermentation. Many fastidious microorganisms are present in fecal microbiota, but some of these could be non-culturable. The stool that was immediately collected and processed for enumeration under strict anerobic conditions contained culturable bacteria of only up to 50% [49]. In addition, the viability of fecal microbiota could be greatly influenced by the subject’s diet, lifestyle, age, and a variety of environmental factors [49]. Reductions in species richness, evenness, and diversity when determined during in vitro fermentation, could be influenced by several limitations. For instance, the competitive effect of the dominant strains [50] and their metabolites, such as short-chain fatty acids [33,51], could inhibit the growth of other microorganisms, as well as the fermentation conditions that have been previously mentioned. The decrease in species richness in the controls may have been due to the microbial starvation caused by an absence of supplementation of SCIINOs in the fermentation medium, as has been reported in a previous study [52]. In contrast, samples collected during SCIINO fermentation by the inoculum obtained from the normal weight subject exhibited similar degrees of evenness and diversity in bacterial species when compared to that of the fecal inoculum (NOC0), while trials conducted using fecal inoculum obtained from the overweight and obese subjects exhibited lower evenness values than those employing their fecal inoculum (Figure 3d). This would indicate that some specific bacteria present in the fecal inoculum of the normal weight subject could uniformly grow in the basal medium without SCIINOs, as has been stated in a previous report [53], while maintaining their initial bacterial evenness and diversity. Moreover, these results were in agreement with those of a previous study, which indicated an ability to effectively utilize SCIINOs (see Section 3.1).

**Figure 4:**
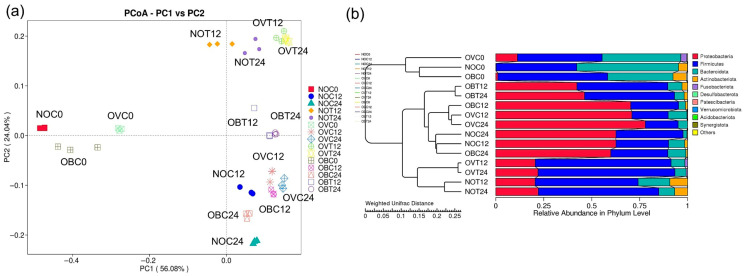
Principal coordinates analysis (PCoA) plot of bacterial communities (**a**) and the UPGMA cluster tree (**b**) based on the weighted UniFrac distance of samples collected during the in vitro fecal fermentation of SCIINOs, and those without SCIINOs (control) using fecal inoculum obtained from normal weight, overweight, and obese subjects.

Beta-diversity is a comparative analysis of microbial community composition for different samples. In this study, differences between analyzed samples were visualized through principal coordinate analysis (PCoA) based on weighted UniFrac distance, which was performed according to the integration of the phylogenetic relationships together with the relative abundance of each sample [54]. This provided insight into how similar or different various microbial communities can be. The PCoA plot exhibited a distinguishable clustering between the bacterial composition of NOC0, OVC0, and OBC0 and those that were treated with SCIINOs (NOT12, NOT24, OVT12, OVT24, OBT12, and OBT24) and without SCIINOs (NOC12, NOC24, OVC12, OVC24, OBC12, and OBC24) (Figure 4a). The results indicate that the fermentation of SCIINOs using different fecal inoculum contributed to significant changes in bacterial composition. The bacterial composition of samples NOT12, NOT24, OVT12, and OVT 24, was clustered in the same group, as opposed to samples OBT12 and OBT24 that were excluded from the group. These results were in accordance with those of the UPGMA cluster tree based on the weighted UniFrac distance (Figure 4b). The samples NOT12, NOT24, OVT12, and OVT24 shared a similar degree of relative abundance at the phylum level, namely Proteobacteria, Firmicutes, and Bacteroidota, while other samples exhibited different values. Remarkably, Actinobacteriota bacteria were found in samples NOT12 and NOT24. According to the high relative abundance of Proteobacteria, as well as the low relative abundance of Firmicutes, samples OBT12 and OBT24 were clearly distinguished from samples NOT12, NOT24, OVT12, and OVT24.

**Figure 5:**
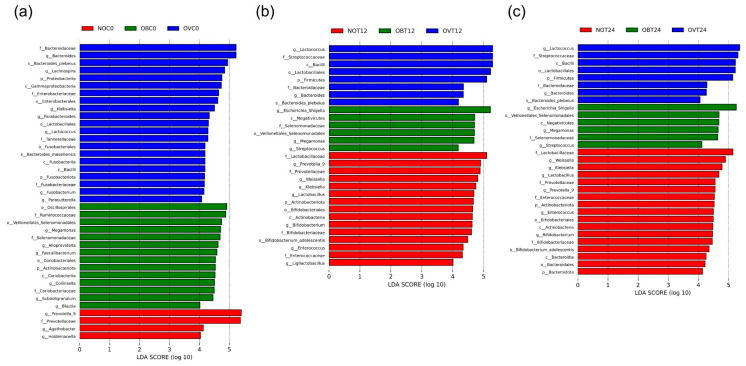
Results of LEfSe (LDA effect size) analysis among analyzed samples; NOC0-OBC0-OVC0 (**a**), NOT12-OBT12-OVT12 (**b**), and NOT24-OBT24-OVT24 (**c**).

Through LEfSe analysis, biomarkers with statistical differences between samples were found. Herein, only the genera associated with the assessed samples were reported. The NOC0 values might be distinguished from OVC0 and OBC0 by genera *Prevotella 9*, *Agathobacter*, and *Holdemanella*. Notably, genera, including *Bacteroides*, *Lachnospira*, *Klebsiella*, *Lactococcus*, *Parabacteroides*, *Fusobacterium*, and *Parasutterella*, were uniquely found in sample OVC0. On the other hand, sample OBC0 possessed genera *Megamonas*, *Alloprevotella*, *Faecalibacterium*, *Collinsella*, *Subdoligranulum*, and *Blautia* (Figure 5a). Certain genera were previously reported to be associated with overweight and obese individuals such as *Megamonas* [55] and *Blautia* [56]. Thus, only the occurrence of *Blautia* in sample OBC0 was determined to be relevant to these previous reports. Because certain limitations exist with regard to the number of subjects, it has been noted that the results may not necessarily be indicators of overweight or obese conditions. Accordingly, these results must be carefully considered. To statistically compare the effects of different fecal inoculum on specific changes in the bacterial community, LEfSe analysis was performed within two groups involving NOT12-OBT12-OVT12 (Figure 5b) and NOT24-OBT24-OVT24 (Figure 5c). Sample NOT12 was unique from others because it consisted of a broad range of genera including *Prevotella 9*, *Weissella*, *Klebsiella*, *Lactobacillus*, *Bifidobacterium*, *Enterococcus*, and *Ligilactobacillus*, while sample OVT12 was associated with specific changes in *Lactococcus* and *Bacteriodes* when compared with the samples NOT12 and OBT12. Accordingly, sample OBT12 was associated with changes in *Escherichia-Shigella*, *Megamonas*, and *Streptococcus*. Notably, similar results were also reported for NOT24-OBT24-OVT24.

**Figure 6:**
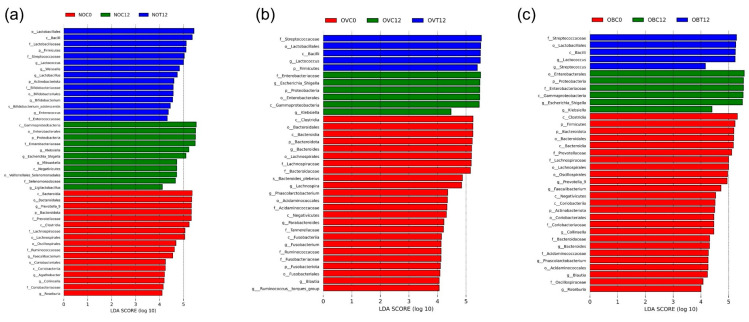
Results of LEfSe (LDA effect size) analysis among analyzed samples; NOC0-NOC12-NOT12 (**a**), OVC0-OVC12-OVT12 (**b**), and OBC0-OBC12-OBT12 (**c**).

On the other hand, to determine the effects of SCIINOs on specific changes in the bacterial community, an LEfSe analysis for NOC0-NOC12-NOT12, OVC0-OVC12-OVT12, and OBC0-OBC12-OBT12 was performed. It was found that, at 12 h of SCIINO fermentation, the bacterial community of normal weight subjects was attributed to changes in the relative abundance values of several bacterial genera including *Lactococcus*, *Weissella*, *Lactobacillus*, *Bifidobacterium* (*B*.) (specifically *B*. *adolescentis*), and *Enterococcus* (Figure 6a). However, the bacterial community of overweight and obese subjects was determined to be responsible for specific changes to *Lactococcus* and *Streptococcus* (Figure 6b,c). Importantly, fermentation without SCIINOs resulted in changes in the relative abundance values of non-specific bacteria, such as *Klebsiella*, *Mitsuokella*, and *Ligilactobacillus* for NOC12, as well as *Escherichia-Shigella* and *Klebsiella* for samples OVC12 and OBC12.

**Figure 7:**
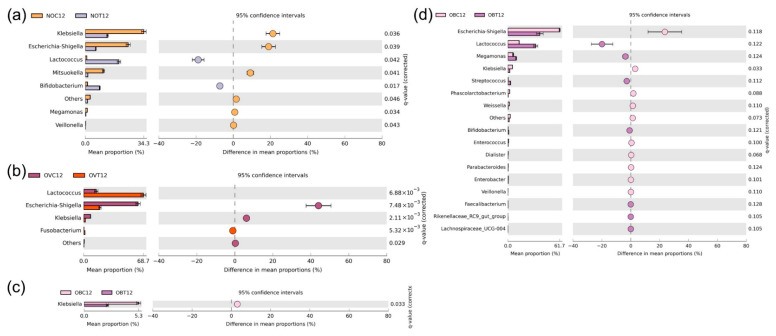
Bacterial relative abundance differences at the genus level were sorted by the most abundant bacterial genera. Significant differences were recorded at *p* < 0.05 in terms of relative abundance between samples; NOC12-NOT12 (**a**), OVC12-OVT12 (**b**), and OBC12-OBT12 (**c**). Significant differences at *p* < 0.20 were recorded in terms of relative abundance between samples OBC12 and OBT12 (**d**).

A *t*-test was used to determine significant variations based on the relative abundance of bacteria between the treatment and control samples, including NOC12-NOT12 (Figure 7a), OVC12-OVT12 (Figure 7b), and OBC12-OBT12 (Figure 7c). The results were sorted by the effect size of each genus that was essentially relevant to the LEfSe results. When fermentation was performed for 12 h, the relative abundance values of *Klebsiella*, *Escherichia-Shigella*, and *Mitsuokella*, that were present in sample NOT12, were lower than those that were present in sample NOC12, while the relative abundance values of *Lactococcus*, *Lactobacillus*, *Bifidobacterium*, and *Enterococcus* were observed to be significantly higher. Comparing the relative abundance of the bacterial community of NOC0-NOT12 (Figure S6a) and NOC0-NOC12 (Figure S6b), it was clear that these bacterial genera were selectively enriched by SCIINOs. Changes in the bacterial composition for OVC12-OVT12 were slightly comparable to those of NOC12-NOT12 in terms of a significant elevation in the relative abundance of *Lactococcus* and a significant decrease in the relative abundance of *Klebsiella*. In addition, the relative abundance of *Fusobacterium*, the bacterium associated with colorectal cancer [57] and a marker for early gut microbial dysbiosis [58], was significantly increased; however, it was then undetected in the sample OVT24. At a significance level of *p* < 0.05, only the relative abundance of *Klebsiella* was significantly decreased in the bacterial community of OBC12 and OBT12 (Figure 7c). This might be related to an inefficient SCIINO utilization profile (see Section 3.1). In further *t*-test analysis at *p* < 0.20 (Figure 7d), the major changes were still associated with an increase in the relative abundance of *Lactococcus* and a decrease in the relative abundance of *Escherichia-Shigella*, which were in accordance with those of NOT12 and OVT12.

Based on a comparison of the bacterial composition of OVC0-OVT12 (Figure S7a) and OBC0-OBT12 (Figure S8a), it is logical to state that SCIINOs could stimulate the abundance of *Lactococcus*. However, the degree of stimulating efficiency was dependent upon the initial fecal microbiota. When comparing the bacterial composition of OVC0-OVC12 (Figure S7b), OVC0-OVT12, OBC0-OBC12 (Figure S8b), and OBC0-OBT12, it was found to be inevitable that the fermentation of SCIINOs could partially elevate the values of abundance of *Escherichia-Shigella* and *Klebsiella*. These have commonly been recognized as pathogens, but their relative abundance values were decreased after SCIINO fermentation. This is especially true for beneficial gut bacteria that were recorded to be promoted by FOSs. Beneficial bacteria, such as *Bifidobacterium* and *Lactobacillus*, are well-adapted to metalizing FOSs. In addition, some lactic acid bacteria capable of producing fructofuranosidases, such as *Lacticaseibacillus* (*Ls.*) *casei*, *Ls*. *paracasei*, *Enterococcus*, and *Streptococcus*, are also able to hydrolyze long-chain FOSs and inulin to form short-chain FOSs, which can then be transported into cells for utilization. Additional strains after lactic acid bacteria have been reported for their ability to grow on FOSs, such as *Escherichia coli*, *Klebsiella*, *Enterobacter*, *Clostridia*, and *Roseburia* [26]; however, their values of relative abundance could also be reduced after FOS fermentation [59]. This statement has relevance to the findings of this study. The enhanced growth of bacterial pathogens during fecal fermentation might take place at the initial stage of fermentation when both pathogens and beneficial microorganisms competitively utilize the fermentable sugars released from SCIINOs. This is one of the general mechanisms of prebiotics and probiotics. It is expected that fecal microbiota containing high microbial composition of beneficial bacteria specific to SCIINO utilization could be considered in major abundance and provide privileged conditions to inhibit bacterial pathogens. This is especially true for the SCIINO fermentation profile using different fecal inoculum of normal weight, overweight, and obese subjects. Interestingly, SCIINOs that can selectively stimulate the growth of *Lactococcus* would be of particular interest. Importantly, it has been reported that *Lactococcus lactis* subsp. *lactis* CAB701 exhibited anti-obesity activity in in vitro and in vivo experiments. It showed inhibitory activity against any differentiation of the adipocyte cell or 3T3-L1 cells. Furthermore, it showed anti-obesity activity in high-fat-diet-induced mice in terms of a reduction in body weight, as well as lowered levels of triglycerides, cholesterol, and low-density lipoprotein cholesterol [60]. Therefore, it was determined that SCIINOs could be used as an alternative anti-obesogenic agent for the prevention and treatment of obesity.

### 3.3. Substrate Specificity of Lactococcus Lactis Toward Neokestose, 1-Kestose, and Nystose

**Figure 8:**
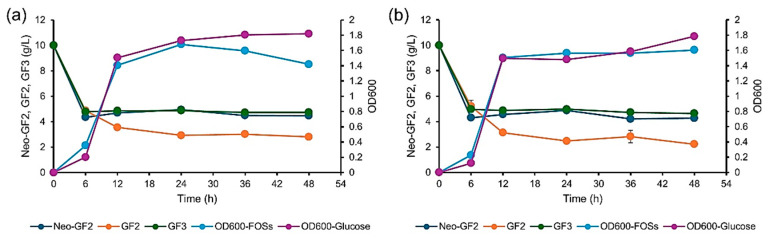
Effect of neokestose, 1-kestose, and nystose mixture on the growth of *Lactococcus lactis* TISTR1401 (**a**) and *Lc*. *lactis* FS38.4 (**b**). Growth of these bacteria on glucose was used as the control.

Remarkably, *Lactococcus* was observed as the dominant bacterial genera that was found in all assessed samples collected from the fermentation of SCIINOs. This is rather notable when compared with previous reports on the fecal fermentation of FOSs. Accordingly, an independent study on the fermentability of FOSs was performed in order to determine which kinds of FOSs are associated with the enrichment of the relative abundance of *Lactococcus*. Two reference strains of *Lc*. *lactis*, including *Lc*. *lactis* TISTR 1401 and *Lc*. *lactis* FS38.4, were used as representative strains to investigate the ability to ferment an FOS mixture containing pure neo-GF_2_, GF_2_, and GF_3_ (each at a level of 10 g/L), while glucose (30 g/L) was used as a positive control. The results are shown in Figure 8. Both strains of *Lc*. *lactis* effectively fermented the FOS mixture in a manner that was comparable to glucose, and they also exhibited a similar pattern for the utilization of each FOS constituent. Neo-GF_2_ and GF_3_ displayed similar fermentation patterns, while their fermentability was less effective than GF_2_. Since there was a trace amount of GF_2_ in SCIINOs, it could be assumed that the selective stimulation of *Lactococcus* during SCIINO fermentation could be attributed to the effects of neo-GF_2_ and GF_3_ rather than those of other longer chain FOSs or GF_2_. The explanation for how *Lc*. *lactis* TISTR 1401 and *Lc*. *lactis* FS38.4 were able to ferment FOSs is still not fully clear, as no β-fructofuranosidase activity was observed in the free-cell supernatant, intact cells, or extracellular fractions. This could have been due to the fact that there may have been too little enzyme activity to be detected. On the other hand, it was suggested that *Lactococcus* sp. metabolizes sugars via a sucrose phosphoenolpyruvate-dependent phosphotransferase system (PTS), which is driven by a set of five genes encoded for sucrose PTS, β-fructofuranosidase, fructokinase, α-glucosidase, and the sucrose operon repressor [61]. This system might be applicable with SCFOSs, as has been reported in the case of *Lactiplantibacillus plantarum* [62]. Moreover, some PTS systems might have expressed specificity to be able to phosphorylate both sucrose and SCFOSs [63]. On the other hand, it has been well documented that FOSs, both inulin and inulin neoseries oligosaccharides, could effectively stimulate the growth of *Bifidobacterium* [5,7,64]. In addition, their ability to promote the growth of other probiotics, including *Lactobacillus*, *Enterococcus*, *Weisella*, and *Streptococcus* [65], has been widely reported. To our knowledge, based on the results of this study, SCIINOs could selectively stimulate the growth of *Lactococcus* in human feces.

### 3.4. Production of Lactic Acid and Short-Chain Fatty Acids

**Figure 9:**
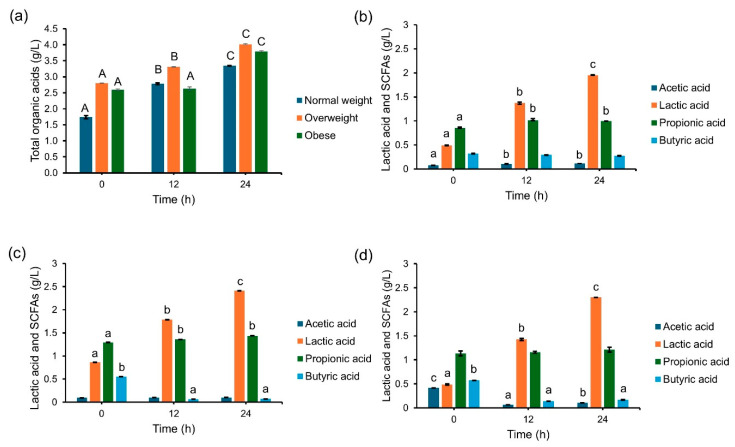
Percentage of total organic acids during the in vitro fecal fermentation process (**a**) and the percentage of each organic acid content during fermentation using the fecal inocula obtained from normal weight (**b**), overweight (**c**), and obese (**d**) subjects. Uppercase letters indicate significant differences (*p* < 0.05) of each subject. Lowercase letters indicate significant differences (*p* < 0.05) of each detectable SCIINOs in different samples.

Intestinal microbiota is the main source of bacteria capable of producing SCFAs through the fermentation of non-digestible carbohydrates and other nutrients [66], while their concentrations can directly reflect the activity of gut microbiota and the produced acidic fermentation environments [67]. In this study, it was found that the pH values of the control samples, NOC12, NOC24, OVC12, OVC24, OBC12, and OBC24, did not differ from those observed at the beginning of the fermentation process, as well as those recorded for the initial inoculum (pH~7). However, this outcome was in contrast with the acidic pH values of the treatment samples, NOT12, NOT24, OVT12, OVT24, OBT12, and OBT24 (pH values ranging from 4.3–4.6). These results are in accordance with a decline in the content of the initial carbohydrates. Notably, these samples were determined for lactic acid and SCFAs, which included acetic acid, propionic acid, butyric acid, and others. Overall, total organic acids during SCIINO fermentation significantly increased from 0 to 24 h (Figure 9a). Lactic acid was substantially produced throughout the fermentation process by different fecal inocula and was dominated as the main organic acid detected. Propionic acid content was detected as the second most abundance of organic acids. It significantly increased during the initial fermentation process using the fecal inoculum of normal weight (Figure 9b) and overweight subjects (Figure 9c), whereas the content was unchanged when compared with the initial content recorded during fermentation using the fecal inoculum of the obese subject (Figure 9d). The residual contents of acetic acid and butyric acid detected during the fermentation of SCIINOs varied depending upon the fecal inocula. Butyric acid content remained at a constant concentration from the initial step until the end of the fermentation process when using the fecal inoculum of the normal weight subject, while its content was significantly decreased from the initial content when fermentation was administered using the fecal inoculum of overweight and obese subjects. When compared with the initial acetic acid contents, a significant increased content was found after fermentation using the fecal inoculum of the normal weight subject. This outcome was in agreement with the determination that the content remained unchanged during fermentation using the fecal inoculum of the overweight subject, while a significant decrease in content was found during fermentation using the fecal inoculum of the obese subject.

Based on the *t*-test analysis of paired samples, NOC0-NOT12 (Figure S6a), OVC0-OVT12 (Figure S7a), and OBC0-OBT12 (Figure S8a), the results indicate the following. There were many reported genera associated with the butyric acid production [68,69,70] found in sample NOT12; however, they showed relatively lower abundance when compared with those of the sample NOC0. These genera sorted by relative abundance included *Facallibacterium*, *Roseburia*, *Eubacterium*, *Coprococcus*, and *Butyricicocus*. It was expected that these bacteria found at low relative abundance contributed to a slight increase in butyric acid content in sample NOT12. Alternatively, it has been reported that an interaction between *Eubacterium*-*Anaerostipes* and *Bifidobacterium*, which was also observed in sample NOT12, could promote butyric acid production [71]. Moreover, *Bifidobacterium* could produce acetic acid, a precursor for butyric acid production [71]. In sample OVT12, only *Faecallibacterium*, a key butyric acid producing bacteria in the gut [72], was detected with a lower relative abundance than that of sample OVT0. This would suggest that this genus might not be a key microorganism exhibiting a crucial role in butyric acid production during SCIINO fermentation. This outcome was in accordance with the decreased level of butyric acid that was present in sample OVT12. Moreover, this finding was in agreement with that which was obtained from sample OBT12, revealing that only *Coprococcus*, a butyric acid-producing microorganism [69], was detected at a low degree of relative abundance. Fermentation of SCIINOs unlikely promoted the formation of butyric acid. Most butyric acid-producing microorganisms are classified as obligated anerobic microorganisms [73], yet the existence of certain inappropriate conditions may limit their growth and can cause low relative abundance, which in turn can reflect a relatively low level of butyric acid content. An increased content of propionic acid when comparing the controls (NOC0 and OVC0) might be associated with *Prevotella 9* for sample NOT12, and *Bacteriodes* for sample OVT12. In contrast, when compared with the control OBC0, no propionic acid-producing microorganisms were detected in sample OBT12, which was in accordance with the constant content of propionic acid detected throughout the fermentation process of SCIINOs. Accordingly, the propionic content remained unchanged throughout the fermentation process. Acetic acid can be produced via the carbohydrate metabolism of heterofermentative lactic acid bacteria [74]. It was found that heterofermentative lactic acid found in samples NOT12 included *Weissella*, *Lactobacillus*, and *Bifidobacterium*. Importantly, *Blautia*, a gut bacterium associated with acetic acid production [56], was detected at a low degree of relative abundance. Heterofermentative lactic acid bacteria was not found in sample OVT12, but low levels of acetic content may have been produced by the *Bacteroides* that were detected in this sample. It has been reported that *Bacteroides* are able to produce acetic acid, succinic acid, lactic acid, and propionic acid [75]. The contents of lactic acid and SCFAs vary depending upon the type of fermentation substrate and the duration time [75]. In the case of sample OBT12, no genus associated with fermentative lactic acid bacteria was present in the sample, but homofermentative lactic acid bacteria, specifically *Lactococcus*, was found as the dominant bacteria. Similar results were also found for samples NOT12 and OVT24. This could be explained by the fact that lactic acid was the main organic acid constituent of SCIINO fermentation. The produced lactic acid can be a precursor for propionic acid or acetic acid [76], as well as butyric acid [77], via lactate producing bacteria such as *Propionibacterium*, *Selenomonas*, *Clostridium propionicum*, *Veillonella*, *Desulfovibrio*, and *Megashaera*. In addition, some strains of *Lactobacillus*, *Streptococcus*, *Lactococcus*, and *Pediococcus* are also capable of producing SCFAs [78,79]. The produced lactic acid and SCFAs have been implicated in inhibitory effects against Gram-negative bacteria and their invasion capability of intestinal cells [80]. This determination was in agreement with our results. Notably, the formation of lactic acid and SCFAs was positively correlated with a significant reduction of *Escherichia-Shigella* and *Klebsiella* in samples NOT12, NOT24, OVT12, OVT24, OBT12, and OBT24. The relative abundance of *Escherichia-Shigella* and *Klebsiella* present in the samples collected after 12 h of fermentation was significantly reduced by 2- to 3-fold when compared with their respective controls. Importantly, this determination corresponded with the fermentation of fructooligosaccharides [81], pomelo pectin [82], and the wild edible mushroom, *Phallus atrovolvatus* [83], in terms of the prebiotics that are able to selectively enrich the growth of *Bifidobacterium*, *Lactobacillus*, *Streptococcus*, and *Bacteroides*, while diminishing the presence of any potential pathogens, specifically *Escherichia-Shigella* and *Klebsiella*. Finally, it could also be concluded that different fecal inocula could directly affect the formation of butyric acid, propionic acid, and acetic acid during SCIINO fermentation. Although they had similar alpha-diversity index results, the microbial composition was found to be different. Notably, the fecal inoculum of the normal weight subject facilitated a more effective fermentation process for SCIINOs and subsequently provided a greater profile of propionic acid, butyric acid, and acetic acid than those of the overweight and obese subjects.

## 4. Conclusions

In this study, an in vitro simulated fermentation model was utilized to assess the fermentability of short-chain inulin and inulin neoseries oligosaccharides (SCIINOs) by fecal inoculum as a natural mixed culture. Three different inocula that were applied in SCIINO fermentation were derived from feces collected from normal weight, overweight, and obese subjects. The SCIINOs consisted of neokestose as the major constituent together with other short-chain inulin-FOSs, namely 1-kestose, nystose, and fructofuranosylnystose. Although there was a limitation in terms of the number of inocula used for SCIINO fermentation, the results revealed that SCIINOs were fermentable by different fecal inocula and provided similar fermentation characteristics in terms of the selective stimulation of *Lactococcus*, as well as any potential effects on a reduction in the abundance of *Escherichia-Shigella* and *Klebsiella*. It seems likely that different fecal inocula might lead to different fermentation characteristics in terms of specificity toward the utilization of SCIINOs and the short-chain fatty acid profile. Therein, the fermentation of SCIINOs by the fecal inoculum obtained from a normal weight subject could stimulate a greater number of beneficial bacterial species than those obtained from overweight and obese subjects. Furthermore, the findings of this research study provide insight into SCIINOs as an alternative to prebiotics that could effectively stimulate the growth of *Lactococcus*. As *Lactococcus* has been suggested to possess anti-obesity activity in in vitro and in vivo experiments, it is expected that SCIINOs could be used as an alternative anti-obesogenic agent for the prevention and treatment of obesity.

## Figures and Tables

**Table 1:** Sample codes established for determination of the effects of SCIINOs on changes in bacterial composition present in feces of normal weight, overweight, and obese individuals.

Subject	Control			Treatment	
	0 h	12 h	24 h	12 h	24 h
NO	NOC0	NOC12	NOC24	NOT12	NOT24
OV	OVC0	OVC12	OVC24	OVT12	OVT24
OB	OBC0	OBC12	OBC24	OBT12	OBT24

The first two letters, NO, OV, and OB, refer to normal weight, overweight, and obese subjects, respectively. Abbreviations T and C presented after NO, OV, and OB indicate samples collected from fecal fermentation with and without SCIINOs, respectively. The numbers presented after each sample indicate the fermentation time that was recorded for any related analysis.

## Data Availability

The original contributions presented in the study are included in the article/Supplementary Material, further inquiries can be directed to the corresponding author.

## Supplementary Materials

The following supporting information can be downloaded at: https://www.mdpi.com/article/10.3390/foods14162804/s1, Table S1: Data preprocessing statistics and quality control; Figure S1: Structure of neokestose, kestose, nystose, and fructofuranosylnystose present in the purified short-chain inulin and inulin neoseries oligosaccharides; Figure S2: Residual fructose content during in vitro fecal fermentation (a) and during fermentation using fecal inoculum obtained from normal weight, overweight, and obese subjects; Figure S3: Relative abundance of bacterial community at the class (a), order (b), and family (c) levels during the in vitro fecal fermentation with (NOT12, NOT24, OVT12, OVT24, OBT12, and OBT24) and without SCIINO (NOC0, NOC12, NOC24, OVC0, OVC12, OVC24, OBC0, OBC12, and OBC24); Figure S4: Relative abundance of the bacterial community of the top 30 genus during in vitro fecal fermentation with SCIINO (NOT12, NOT24, OVT12, OVT24, OBT12, and OBT24) and without SCIINO (NOC0, NOC12, NOC24, OVC0, OVC12, OVC24, OBC0, OBC12, and OBC24); Figure S5: Box plots presenting the Good’s coverage index of bacterial communities in samples collected during in vitro fecal fermentation of SCIINO, and those without SCIINO (control) using fecal inoculum obtained from normal weight, overweight, and obese subjects; Figure S6: Bacterial relative abundance differences (*p* < 0.05) in comparisons made between samples; NOC0-NOT12 (a) and NOC0-NOC12 (b); Figure S7: Bacterial relative abundance differences (*p* < 0.05) in comparisons made between samples; OVC0-OVT12 (a) and OVC0-OVC12 (b); Figure S8: Bacterial relative abundance differences (*p* < 0.05) in comparisons made between samples; OBC0-OBT12 (a) and OBC0-OBC12 (b).
