# Supplementary material for: In Vitro Fermentation Characteristics of Purified Short-Chain Inulin and Inulin Neoseries Oligosaccharides Produced from Red Onions
Source: Foods. 2025 Aug 13;14(16):2804. doi: 10.3390/foods14162804 (PMC12385300; doi:10.3390/foods14162804)
Supplement: Supplementary file 1 [file foods-14-02804-s001.zip › foods-3774929-supplementary.pdf]

# Supplementary Materials

***In vitro* fermentation characteristics of purified short-chain inulin and inulin neoseries oligosaccharides produced from red onions**

Jirat Wongsanittayarak [1,2], Apinun Kanpiengjai [2,*], Nalapat Leangnim [2,3] , Supachawadee Soyprasert [1,2], Kridsada Unban [4], Saisamorn Lumyong [5,6], Chartchai Khanongnuch [7], Pairote Wongputtisin [8]

[1] Program in Biotechnology, Multidisciplinary and Interdisciplinary School, Chiang Mai University, Chiang Mai, 50200, Thailand

[2] Division of Biochemistry and Biochemical Innovation, Department of Chemistry, Faculty of Science, Chiang Mai University, Chiang Mai 50200, Thailand

[3] Office of Research Administration, Chiang Mai University, Chiang Mai 50200, Thailand

[4] Division of Food Science and Technology, Faculty of Agro-industry, Chiang Mai University, Chiang Mai 50100, Thailand

[5] Department of Biology, Faculty of Science, Chiang Mai University, Chiang Mai 50200, Thailand

[6] Academy of Science, The Royal Society of Thailand, Bangkok 10300, Thailand

[7] Center of Excellence in Agricultural Innovation for Graduate Entrepreneur, Maejo University, Chiang Mai 50290, Thailand

[8] Program in Biotechnology, Faculty of Science, Maejo University, Chiang Mai 50290, Thailand

***Corresponding author:** Apinun Kanpiengjai

**Corresponding author email:** apinun.k@cmu.ac.th, ak.apinun@gmail.com

**Table S1** Data preprocessing statistics and quality control.

| Sample | RawPE | Combined | Qualified | Nochime | Base(nt) | Avglen(nt) |
|---|---|---|---|---|---|---|
| NOC0R1 | 203467 | 201578 | 198468 | 167081 | 69616970 | 416.67 |
| NOC0R2 | 218417 | 217272 | 214080 | 179310 | 74859713 | 417.49 |
| NOC0R3 | 202516 | 201369 | 198510 | 165503 | 68977183 | 416.77 |
| NOC12R1 | 205227 | 203934 | 200571 | 166757 | 71396695 | 428.15 |
| NOC12R2 | 202911 | 201651 | 198258 | 162470 | 69599893 | 428.39 |
| NOC12R3 | 205054 | 203838 | 200433 | 173128 | 74166589 | 428.39 |
| NOC24R1 | 202472 | 201369 | 198179 | 162175 | 69546616 | 428.84 |
| NOC24R2 | 202840 | 201787 | 198487 | 166557 | 71423881 | 428.83 |
| NOC24R3 | 157327 | 156502 | 153790 | 132200 | 56693218 | 428.84 |
| NOT12R1 | 219308 | 217814 | 213571 | 183472 | 78288126 | 426.7 |
| NOT12R2 | 214128 | 212825 | 209068 | 163850 | 69849839 | 426.3 |
| NOT12R3 | 204640 | 203327 | 199666 | 153954 | 65621621 | 426.24 |
| NOT24R1 | 219312 | 217956 | 213964 | 175900 | 75201486 | 427.52 |
| NOT24R2 | 219079 | 217555 | 213284 | 169081 | 72264910 | 427.4 |
| NOT24R3 | 218699 | 217338 | 213430 | 185821 | 79351069 | 427.03 |
| OVC0R1 | 174199 | 173001 | 170287 | 143214 | 59764454 | 417.31 |
| OVC0R2 | 212385 | 211018 | 207689 | 154718 | 64669910 | 417.99 |
| OVC0R3 | 202573 | 201386 | 198648 | 135438 | 56602680 | 417.92 |
| OVC12R1 | 203388 | 202148 | 198831 | 185806 | 79508240 | 427.91 |
| OVC12R2 | 203853 | 202655 | 199353 | 181953 | 77834910 | 427.77 |
| OVC12R3 | 203423 | 202251 | 199078 | 178906 | 76557921 | 427.92 |
| OVC24R1 | 203857 | 202553 | 199079 | 185932 | 79690206 | 428.6 |
| OVC24R2 | 204318 | 203108 | 199786 | 184758 | 79194425 | 428.64 |
| OVC24R3 | 177361 | 176336 | 173361 | 161042 | 69039491 | 428.7 |
| OVT12R1 | 214706 | 212992 | 208485 | 194162 | 83127314 | 428.13 |
| OVT12R2 | 202099 | 200502 | 196328 | 189682 | 81165781 | 427.9 |
| OVT12R3 | 203909 | 202267 | 197723 | 181775 | 77797443 | 427.99 |

| | | | | | | |
|---|---|---|---|---|---|---|
| OVT24R1 | 204684 | 203092 | 198827 | 188418 | 80710818 | 428.36 |
| OVT24R2 | 211646 | 209958 | 205494 | 192633 | 82534743 | 428.46 |
| OVT24R3 | 211142 | 209382 | 205075 | 195054 | 83559475 | 428.39 |
| OBC0R1 | 180048 | 179122 | 176404 | 142173 | 59113762 | 415.79 |
| OBC0R2 | 204711 | 203567 | 200486 | 160054 | 66663088 | 416.5 |
| OBC0R3 | 202426 | 201342 | 198442 | 171442 | 71000694 | 414.14 |
| OBC12R1 | 202009 | 200916 | 197668 | 184367 | 78998741 | 428.49 |
| OBC12R2 | 209952 | 208561 | 204911 | 188081 | 80599383 | 428.54 |
| OBC12R3 | 205407 | 204279 | 201069 | 184237 | 78947560 | 428.51 |
| OBC24R1 | 204313 | 203046 | 199788 | 177419 | 75986825 | 428.29 |
| OBC24R2 | 203401 | 202109 | 198872 | 170783 | 73144977 | 428.29 |
| OBC24R3 | 204732 | 203370 | 199754 | 173789 | 74438445 | 428.33 |
| OBT12R1 | 209094 | 207739 | 204074 | 186789 | 79884604 | 427.67 |
| OBT12R2 | 216859 | 215289 | 210962 | 190317 | 81448439 | 427.96 |
| OBT12R3 | 213200 | 211727 | 207928 | 195915 | 83863441 | 428.06 |
| OBT24R1 | 203473 | 202086 | 198449 | 186499 | 79872886 | 428.28 |
| OBT24R2 | 207853 | 206470 | 202669 | 192399 | 82416907 | 428.36 |
| OBT24R3 | 203991 | 202579 | 198857 | 182850 | 78307912 | 428.26 |

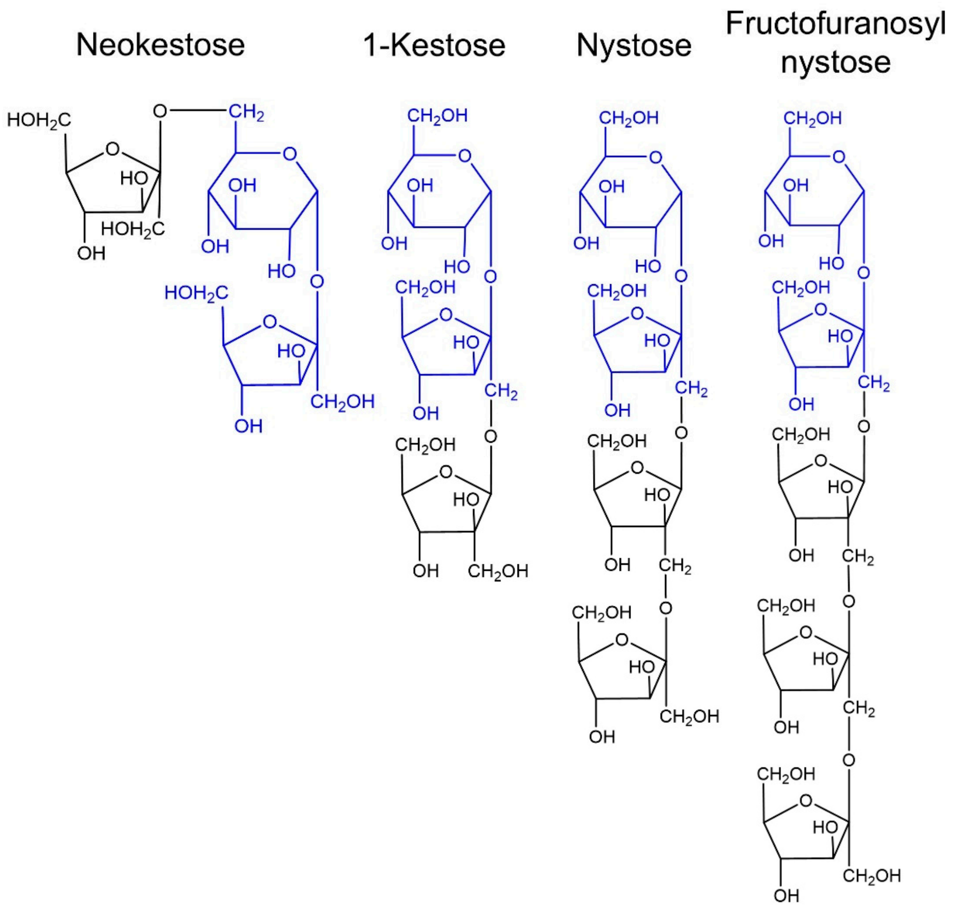


**Figure S1** Structure of neokestose, kestose, nystose, and fructofuranosylnystose present in the purified short-chain inulin and inulin neoseries oligosaccharides.

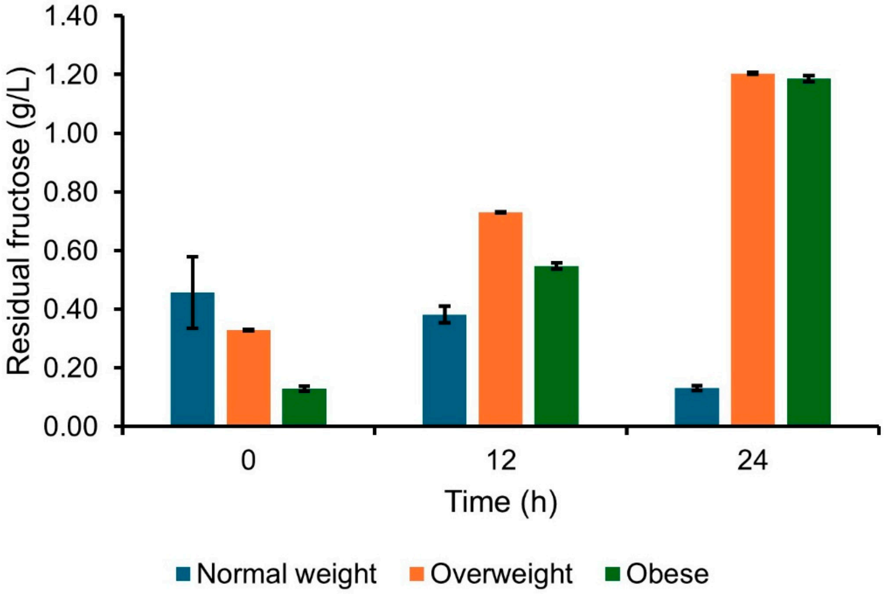


**Figure S2** Residual fructose content during *in vitro* fecal fermentation (a) and during fermentation using fecal inoculum obtained from normal weight, overweight, and obese subjects.

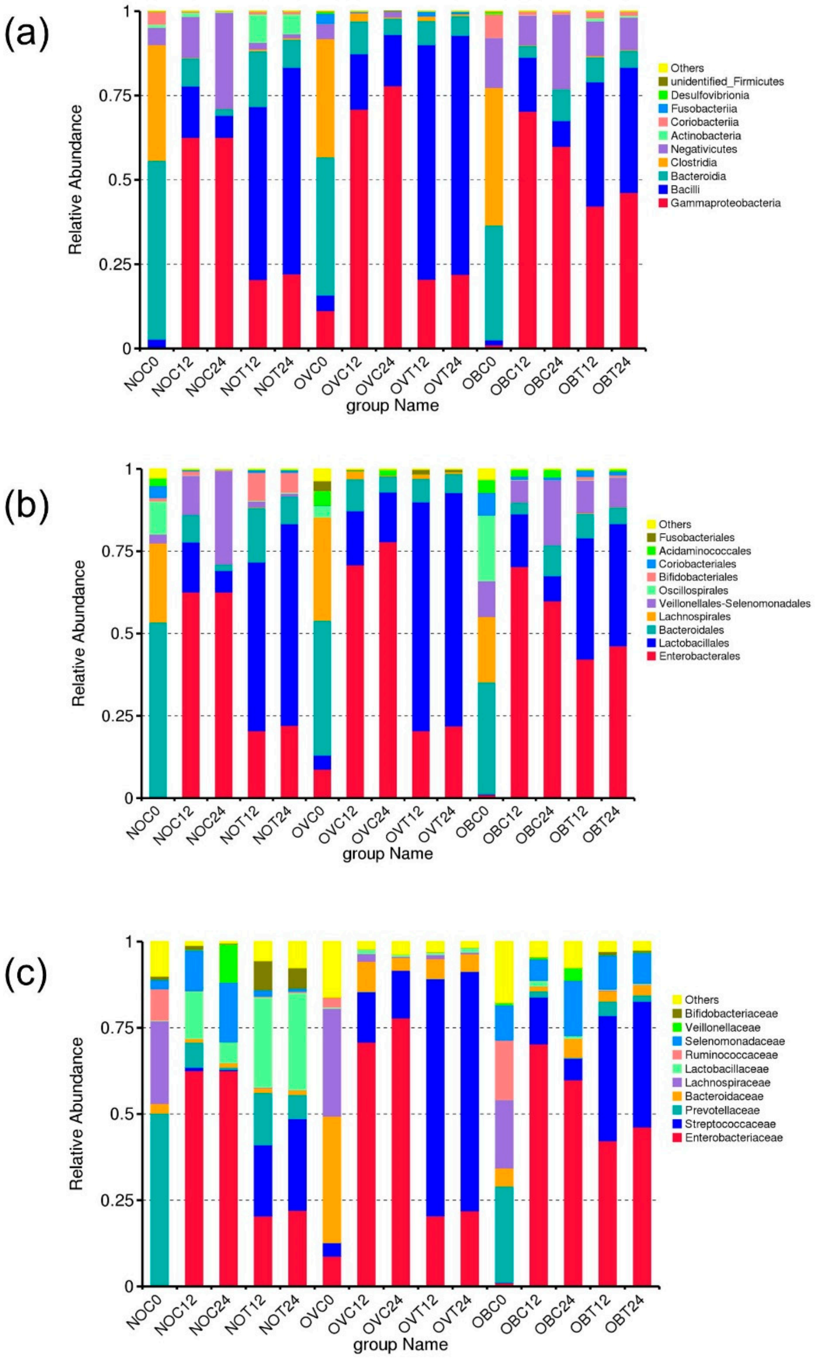


**Figure S3** Relative abundance of bacterial community at the class (a), order (b), and family (c) levels during the *in vitro* fecal fermentation with (NOT12, NOT24, OVT12, OVT24, OBT12, and OBT24) and without SCIINO (NOC0, NOC12, NOC24, OVC0, OVC12, OVC24, OBC0, OBC12, and OBC24).

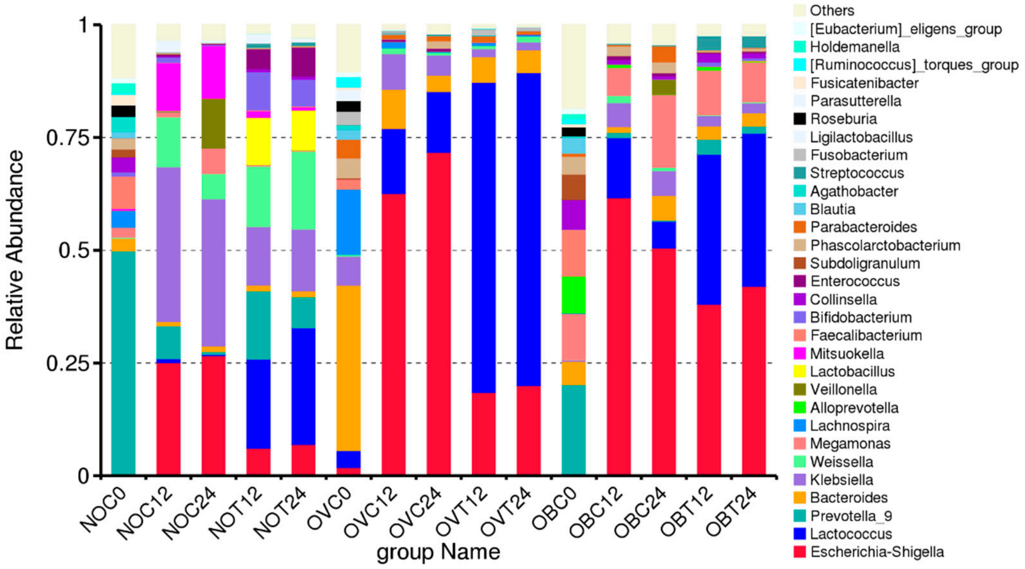


**Figure S4** Relative abundance of the bacterial community of the top 30 genus during *in vitro* fecal fermentation with SCIINO (NOT12, NOT24, OVT12, OVT24, OBT12, and OBT24) and without SCIINO (NOC0, NOC12, NOC24, OVC0, OVC12, OVC24, OBC0, OBC12, and OBC24).

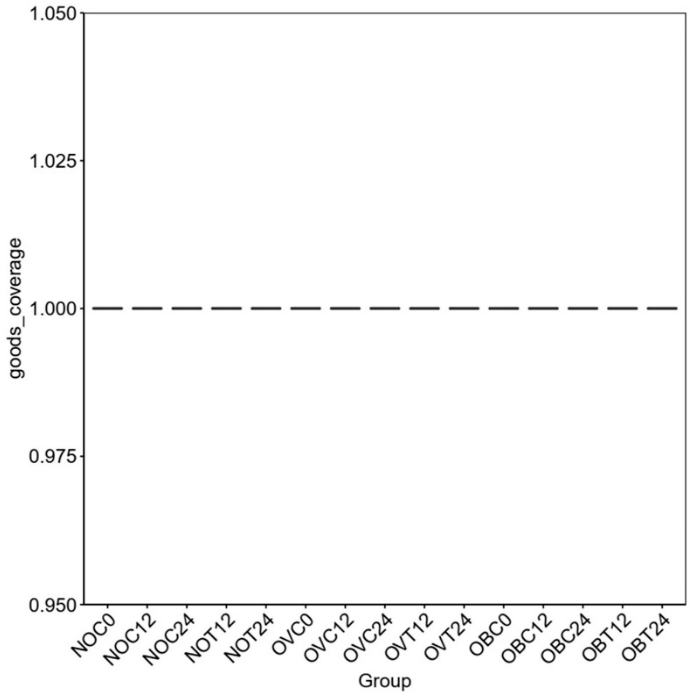


**Figure S5** Box plots presenting the Good's coverage index of bacterial communities in samples collected during *in vitro* fecal fermentation of SCIINO, and those without SCIINO (control) using fecal inoculum obtained from normal weight, overweight, and obese subjects.

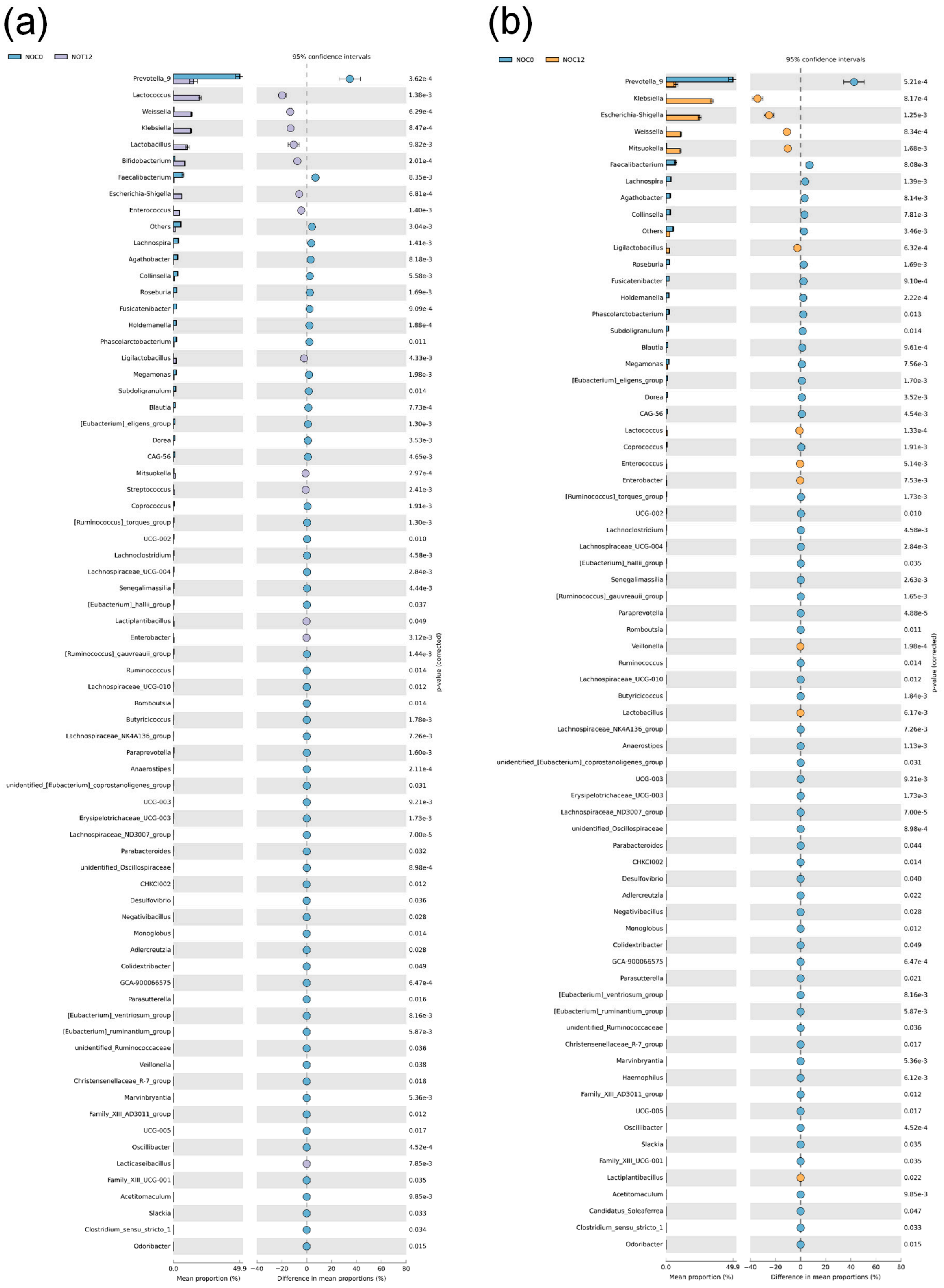


**Figure S6** Bacterial relative abundance differences ($p < 0.05$) in comparisons made between samples; NOC0-NOT12 (a) and NOC0-NOC12 (b).

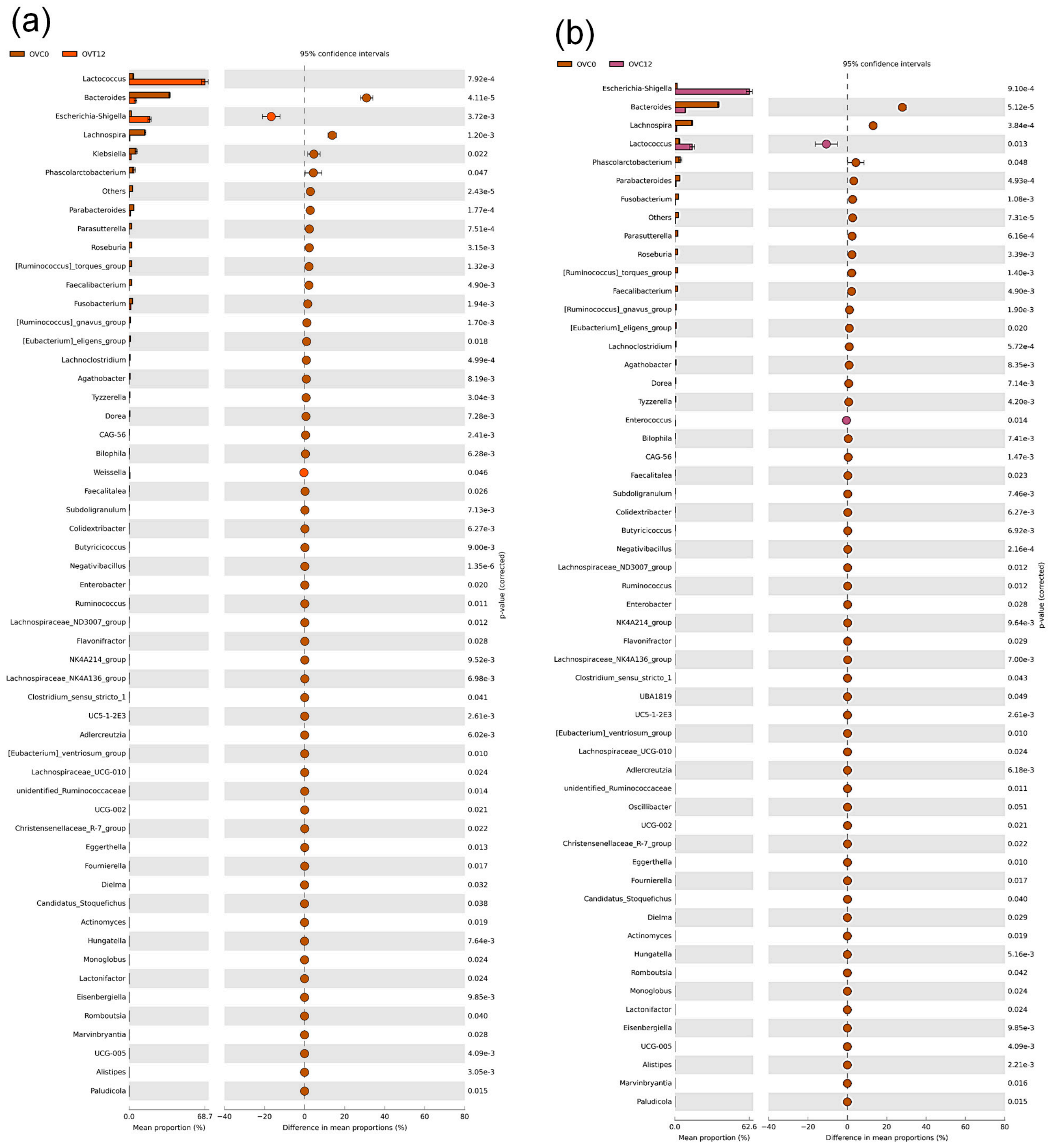


**Figure S7** Bacterial relative abundance differences ($p < 0.05$) in comparisons made between samples; OVC0-OVT12 (a) and OVC0-OVC12 (b).

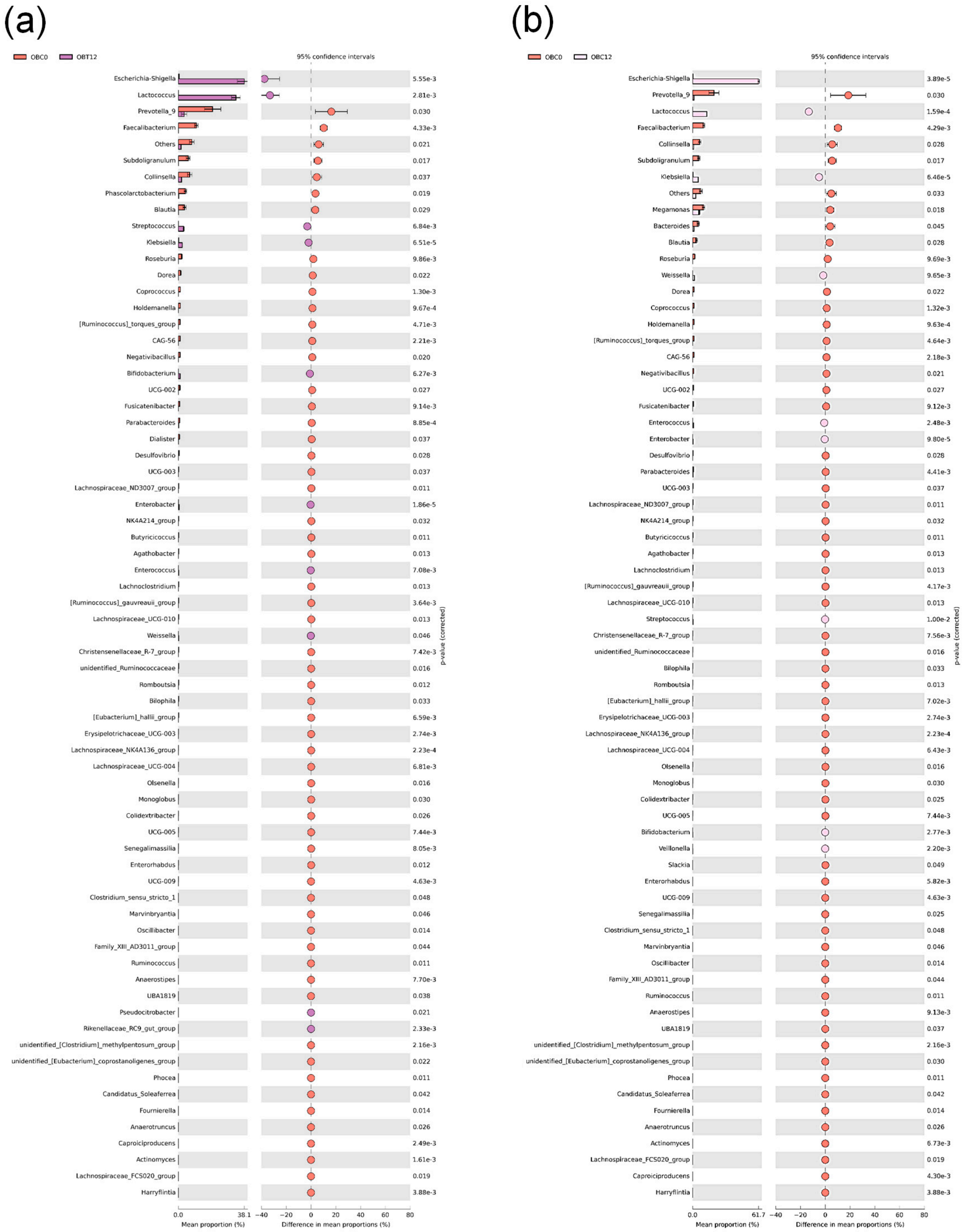


**Figure S8** Bacterial relative abundance differences ($p < 0.05$) in comparisons made between samples; OBC0-OBT12 (a) and OBC0-OBC12 (b).
